# Predictive value of serum chitinase 3-like 1 for dysfunctional autogenous arteriovenous fistulas in patients with chronic kidney disease stage G5

**DOI:** 10.1016/j.clinsp.2025.100852

**Published:** 2025-12-30

**Authors:** You Wen Lin, Qing Zhang, Ying Sheng Xu, Ting Qu

**Affiliations:** a Department of Nephrology, Qinghai Provincial People’s Hospital, Xining City, Qinghai Province, China; b Department of Nephrology, Ezhou Central Hospital, Ezhou City, Hubei Province, China; c Department of Clinical Nutrition, Ezhou Central Hospital, Ezhou City, Hubei Province, China; d Department of Nephrology, People’s Hospital of Chongqing Liang Jiang New Area, Chongqing City, China

**Keywords:** Chitinase 3-like-1, Chronic kidney disease stage G5, Arteriovenous fistula, Failure, Prediction

## Abstract

**Objective:**

Arteriovenous Fistula (AVF) is the preferred vascular access for patients with Chronic Kidney Disease Stage G5 (CKDG5). Normal AVF function is an important prerequisite for hemodialysis, and there are few reports of studies predicting the factors affecting dysfunctional AVF. The aim of this study was to investigate the expression of Chitosanase-3-Like protein-1 (CHI3L1) in AVFs from patients with end-stage renal disease and to analyze the potential mechanism of its role in AVF dysfunction.

**Methods:**

CKDG5 patients who underwent AVF surgery at our institution from January 2020 to September 2023 were prospectively collected. The general clinical data and laboratory data were collected in detail, and the occurrence of postoperative dysfunctional AVF was recorded. Then, the optimal diagnostic threshold of serum CHI3L1 was observed by the Receiver Operating Characteristic (ROC) curve. Kaplan-Meier survival curve and Log-rank test were used for survival analysis. Multi-factor backward logic analysis was used to screen baseline clinical factors and construct prediction Model 1. Subsequently, serum CHI3L1 was combined with baseline clinical factors to construct Prediction Model 2. The predictive value of the two models was evaluated by the ROC curve and the Area Under the Curve (AUC).

**Results:**

A total of 152 CKDG5 patients who underwent AVF surgery were continuously included. They were divided into a normal AVF group (*n* = 64) and a dysfunctional AVF group (*n* = 88). Gender, age, body mass index, primary disease, diameter of cephalic vein, diameter of radial artery, follow-up time, and levels of Ca, K, total cholesterol, triglycerides, high-density lipoprotein, low-density lipoprotein, white blood cell count, platelets, fasting blood glucose, and hemoglobin in both groups were not statistically significant between the two groups (*p* > 0.05). Systolic blood pressure, diastolic blood pressure, parathyroid hormone, serum albumin, and CHI3L1 were increased, whereas serum phosphorus level was decreased in CKDG5 patients with dysfunctional AVF (*p* < 0.05). The AUC of serum CHI3L1 was 0.797 (95% CI 0.728‒0.866, *p* < 0.001), the optimal cut-off value was 148.0 ng/mL, the sensitivity was 62.5%, and the specificity was 84.38%. The primary fluency rate in the high serum CHI3L1 level group was significantly lower than that in the low serum CHI3L1 level group (*p* < 0.001). Multifactorial logistic regression analysis showed that elevated serum CHI3L1 was an independent risk factor for postoperative dysfunctional AVF. The predictive value of the CHI3L1-containing predictive Model 2 was higher, with a net benefit at threshold probabilities of 0.2‒0.9 for its clinical decision curve.

**Conclusion:**

This study proposes a risk prediction model of serum CHI3L1 combined with clinical risk factors, which can be utilized to make a preliminary prediction of the risk of dysfunctional AVF more easily, and thereafter to identify and intervene in high-risk patients early.

## Highlights

• Uterine carcinosarcoma is treated with no histology-based management.
• Heterologous sarcomatous component has a negative impact on overall survival.
• First study to report a negative prognostic impact in FIGO I-IVA in Latin America.
• There is a need to reconsider the role of the sarcomatous component to tailor treatment.

## Introduction

Chronic Kidney Disease Stage G5 (CKDG5) has high morbidity and mortality rates.^1^ Clinicians often use hemodialysis, kidney transplantation, peritoneal dialysis and other treatment modalities.^2^ In China, there has been a swift rise in Maintenance Hemodialysis (MHD) patients, driven by the growing number of CKDG5 patients and improvements in hemodialysis techniques.^3^ Appropriate vascular access is the premise of MHD. Arteriovenous Fistula (AVF) is the preferred vascular access for MHD at present.^4^^,^^5^ However, in clinical practice, dysfunctional AVF may be caused by various reasons, resulting in lumen stenosis or occlusion, thereby reducing the blood flow of the vascular access and affecting the therapeutic effect of MHD.^6^ In the present day, color Doppler ultrasound is a common method for diagnosing AVF function.^7^^,^^8^ However, due to the long interval, it cannot reflect the function of AVF in time. As a result, a simple, efficient, and timely way of monitoring AVF function is needed.

Chitinase-3-Like protein-1 (CHI3L1) is a secreted glycoprotein.^9^ Elevated baseline serum CHI3L1 levels are independently associated with a higher risk of early forearm dysfunctional AVF, and the association between baseline CHI3L1 concentration and dysfunctional AVF is stable and robust.^10^ In addition, dysfunctional AVF may be associated with CHI3L1 in vascular tissue.^11^ Therefore, it is speculated that CHI3L1 may be involved in fistula stenosis and dysfunction. However, few studies have been conducted on the predictive value of CHI3L1 on dysfunctional AVF in CKDG5 patients, and its potential mechanism and clinical application have not been fully clarified. Based on this, this study analyzed the correlation between serum CHI3L1 level and dysfunctional AVF in patients with CKDG5 undergoing AVF surgery, with the aim of exploring the predictive value of CHI3L1 on dysfunctional AVF, and providing new theoretical basis and practical guidance for the early identification of high-risk patients in the clinic and the formulation of precise intervention strategies.

## Materials and methods

### Patients

From January 2020 to September 2023, patients with CKD in stage V who underwent AVF surgery for the first time and were followed up regularly in Qinghai Provincial People’s Hospital were selected as research subjects.

Inclusion criteria: 1) Patients aged ≥ 18-years at the time of AVF establishment; Patients with radial artery and cephalic vein diameter > 2 mm based on preoperative Doppler ultrasound and those without special surgical contraindications; 2) Patients undergoing first AVF surgeries (radial artery-cephalic vein) and receiving subsequent MHD; 3) Patients with AVF as the only vascular access of MHD; 4) Patients receiving regular MHD more than 3-months, 3 times a week; 5) Patients with complete clinical data.

Exclusion criteria: 1) Patients aged < 18-years; 2) Patients with previous fistula resection or reconstruction; 3) Patients with graft fistula; 4) Patients who were transferred to peritoneal dialysis or received renal transplantation; 5) Patients with tumor or severe cardiovascular and cerebrovascular diseases; 6) Patients with abnormal liver function, infection or blood system diseases; 7) Patients with long-term use of anticoagulant and antiplatelet drugs; 8) Pregnant or lactating female patients; 9) Patients who cannot come to the hospital on time for follow-up. This study was conducted following the STROBE statement. The present study was approved by the Ethics Committee of Qinghai Provincial People’s Hospital (n° 201806QH2544), and written informed consent was provided by all patients prior to the study start.

### AVF surgery

The physical examination and ultrasound examination of the blood vessels at the surgical site were performed by the surgeon for the patients undergoing AVF surgery. The inner diameter of the vein and artery and the arterial blood flow velocity were measured by ultrasound. End-to-side forearm cephalic vein-radial artery anastomosis was performed on patients without contraindications under local anesthesia. Ultrasound examination of the radial artery and cephalic vein was repeated after surgery and before MHD. MHD was started in patients with non-stenosis. The surgery was performed by the same physician.

### Diagnostic criteria of dysfunctional AVF

Dysfunctional AVF can be defined as the presence of one of the following three items^12^: 1) There was no palpable tremor, and the auscultation vascular murmur was weak or absent; 2) The actual blood flow during MHD was less than 200 mL/min; 3) Ultrasound examination showed that there may be vascular intimal hyperplasia or thrombosis. According to the clinical diagnostic criteria of dysfunctional AVF, the patients were classified with dysfunctional AVF and normal AVF, respectively.

### Data collection

1) Basic data, including age, gender, Body Mass Index (BMI), Systolic Blood Pressure (SBP), Diastolic Blood Pressure (DBP) before MHD, and the presence of primary diseases, were collected.

2) The diameter of the cephalic vein and the radial artery were measured by color Doppler ultrasound before MHD. The patient's cephalic vein diameter and radial artery diameter were detected using a portable color Doppler ultrasound instrument (Sonoson, USA, model: M-Turbo), which was done by 2 specially trained ultrasonographers with more than 8-years of experience in vascular ultrasound, and the parameters were averaged on 3 consecutive occasions. Patients received routine fasting blood tests at least 3 months after initial dialysis. Fasting elbow venous blood (5 mL/tube) was collected one day before AVF surgery (for patients with normal AVF, blood sampling was performed before the study), a total of two tubes. One tube was anticoagulated with ethylenediamine tetraacetic acid and centrifuged at 1000 r/min for 15 min. The upper serum was taken and stored at −80 °C to detect CHI3L1 in serum. The other tube was sent for blood routine (blood routine analyzer, ABX MICROS 60) and biochemical indicators (automatic biochemical detector, Toshiba, TBA-120FR). Parathyroid Hormone (PTH), Calcium (Ca), Phosphorus (P), Potassium (K), Uric Acid (UA), Total Cholesterol (TC), Triglyceride (TG), Low Density Lipoprotein (LDL), High Density Lipoprotein (HDL), Albumin (ALB), White Blood Cell Count (WBC), Platelet (PLT), Fibrinogen (FG), Fasting blood Glucose (FBG), and Hemoglobin (HB) were recorded.

3) Serum CHI3L1 level of all patients was detected by enzyme-linked immunosorbent assay kit (Solarbio, Beijing, China).

### Follow-up

The patients were followed up from the first establishment of AVF until the first discovery of dysfunctional AVF. In the case of patients without dysfunctional AVF, the follow-up ended on September 1, 2023.

### Statistical analysis

SPSS 25.0 statistical software was used for data analysis. Data normality was checked by the Shapiro-Wilk test. The measurement data satisfying the normal distribution were expressed as mean ± standard deviation, and the *t*-test was used for comparison between groups. Non-parametric test was used for non-normally distributed measurement data and count data. The measurement data were expressed as median and interquartile range [M (Q1, Q3)], and the Mann-Whitney *U* rank sum test was used to compare the differences between groups. The enumeration data were expressed as examples and percentages n ( %), and the differences were analyzed by the Chi-Square test or Fisher's exact test. The test of statistical significance was a two-sided test, and *p* < 0.05 was defined as the difference was statistically significant. The predictive ability of serum CHI3L1 for dysfunctional AVF in CKDG5 patients was analyzed by Receiver Operating Characteristic curves (ROC) analysis, and the Area Under the Curve (AUC), optimal threshold at the maximum of the Youden index, sensitivity, and specificity were calculated. Kaplan-Meier survival curve was drawn, and the Log-rank test was used to compare the primary fluency rate between groups. Stepwise backward logistic regression was used to analyze the variables with statistical significance (*p* < 0.05) in univariate analysis. Significance testing for the logistic regression model was performed using the Wald test. Continuous variables were directly input into multivariate logistic regression to screen out independent influencing factors. The linear relationship between the screened independent influences was investigated using the Variance Inflation Factor (VIF). The predictive value of the model was evaluated by ROC and AUC. The calibration curve, nomogram and Decision Curve Analysis (DCA) curve were drawn with the software package 4.0.5 of R language. This new prediction strategy, DCA, is superior to the ROC curve, utilizing the net benefit rate as the ordinate and the threshold probability as the abscissa. Besides the x-axis and y-axis, there are two reference lines known as invalid lines. The None line indicates that no clinical interventions are applied to any patients, while the All line signifies that clinical interventions are implemented. To be considered high-quality, a prediction model must have a net return rate that exceeds the two invalid lines within a defined threshold range or at least reaches a certain threshold. The optimized calibration curve and Hosmer-Lemeshow test (HL test) were used to evaluate the calibration performance of the model. *p* > 0.05 indicated good fitness, indicating that there was no significant difference between the predicted value and the true value. The nomogram was used to evaluate the contribution of each independent variable to the outcome event. Data were plotted using GraphPad Prism 8. *p* < 0.05 was considered statistically significant.

## Results

### General baseline data

**Fig. 1:**
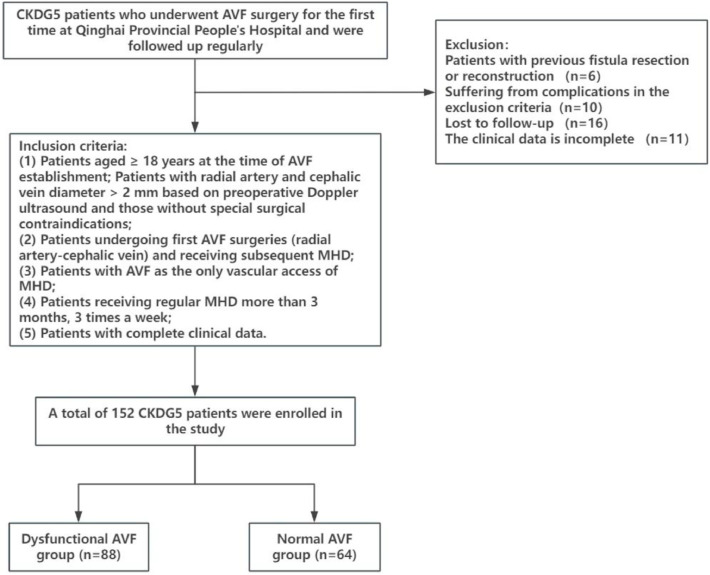
Flowchart of patients' inclusion and exclusion criteria.

A total of 152 patients were included in this study, including 88 patients with dysfunctional AVF and 64 patients with normal AVF. The patient inclusion and exclusion flowchart is shown in Fig. 1. The median follow-up was 19-months.^14^, ^15^, ^16^, ^17^, ^18^, ^19^, ^20^, ^21^, ^22^, ^23^, ^24^, ^25^, ^26^, ^27^ There was no significant difference in gender, age, BMI, primary disease, cephalic vein diameter, radial artery diameter, follow-up time, Ca, K, TC, TG, HDL, LDL, WBC, PLT, FBG and HB levels between the two groups (*p* > 0.05). SBP, DBP, PTH, ALB, and CHI3L1 in the dysfunctional AVF group were significantly higher than those in the normal AVF group (*p* < 0.05). Compared with the normal AVF group, the P level of the dysfunctional AVF group was lower (*p* < 0.05) (Table 1).

**Table 1:** Comparison of clinical data and serum CHI3L1 level between the two groups of patients.

Data	Dysfunctional AVF group (*n* = 88)	Normal AVF group (*n* = 64)	p
Age	54.37 ± 13.08	53.44 ± 12.42	0.659
Gender (male/female)	48/40	34/30	0.871
Body mass index	23.31 ± 3.24	23.60 ± 3.55	0.602
SBP (mmHg)	148.16 ± 17.34	140.74 ± 18.91	0.0132
DBP (mmHg)	93.08 ± 12.43	87.60 ± 13.27	0.01
Primary diseases			0.835
Hypertension	29	20	
Diabetes	22	19	
Cardiovascular diseases	7	6	
Nephrotic syndrome	26	18	
Systemic lupus erythematosus	4	1	
Cephalic vein diameter (cm)	2.31 ± 0.42	2.29 ± 0.41	0.77
Radial artery diameter (cm)	2.10 ± 0.34	2.16 ± 0.38	0.308
Follow-up (months)	31 (10–43)	30.5 (11–48)	0.536
Parathyroid hormone (pg/mL)	393.62 ± 114.26	338.51 ± 92.82	0.104
Blood calcium (mmoL/L)	2.19 ± 0.30	2.20 ± 0.27	0.833
Se rum phosphorus (mmoL/L)	2.2 ± 0.45	2.02 ± 0.53	0.002
Serum potassium (mmoL/L)	4.83 ± 0.83	4.71 ± 0.77	0.366
Blood uric acid (umoL/L)	465.27 ± 95.80	402.75 ± 85.61	<0.001
Total cholesterol (mmoL/L)	4.26 ± 1.32	4.24 ± 1.35	0.927
Triglyceride (mmoL/L)	1.76 ± 0.43	1.71 ± 0.49	0.506
High density lipoprotein (mmoL/L)	1.11 ± 0.28	1.16 ± 0.35	0.33
Low density lipoprotein (mmoL/L)	2.32 ± 0.72	2.43 ± 0.78	0.371
Serum albumin (g/L)	38.93 ± 4.89	36.93 ± 5.65	0.021
White blood cells (× 109/L)	6.57 ± 2.01	6.18 ± 1.86	0.225
Platelets (× 109/L)	202.50 ± 64.29	203.42 ± 61.82	0.93
Fibrinogen (g/L)	4.23 ± 0.94	3.75 ± 0.86	0.012
Fasting blood glucose (mmoL/L)	6.63 ± 2.64	7.2 ± 3.18	0.23
Hemoglobin (g/L)	105.05 ± 15.49	107.84 ± 18.24	0.311
CHI3L1 (ng/mL)	158.47 ± 37.15	117.25 ± 31.24	<0.001

### ROC curve of serum CHI3L1 in predicting dysfunctional AVF in CKDG5 patients

**Fig. 2:**
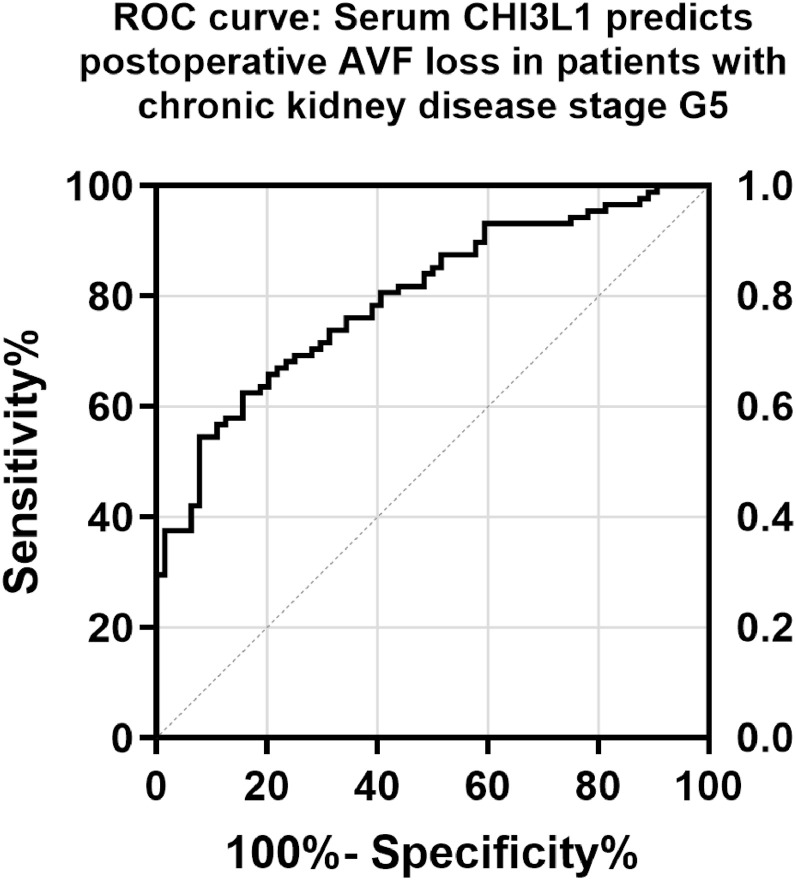
ROC curve of serum CHI3L1 in predicting postoperative dysfunctional AVF in CKDG5 patients.

According to the follow-up results, the ROC curve was drawn. The AUC of serum CHI3L1 was 0.797, the maximum Youden index was 0.469, the optimal cut-off value was 148.0, the sensitivity was 62.50 %, and the specificity was 84.38 % (Table 2, Fig. 2).

**Table 2:** ROC analysis of serum CHI3L1 in predicting postoperative dysfunctional AVF in patients with CKDG5.

Indicator	AUC	Cut-off value	95 % CI	Sensitivity	Specificity	p
Serum CHI3L1	0.797	148.0 (ng/mL)	0.728‒0.866	62.50 %	84.38 %	<0.001

### Survival curves of CKDG5 patients with different CHI3L1 levels

**Fig. 3:**
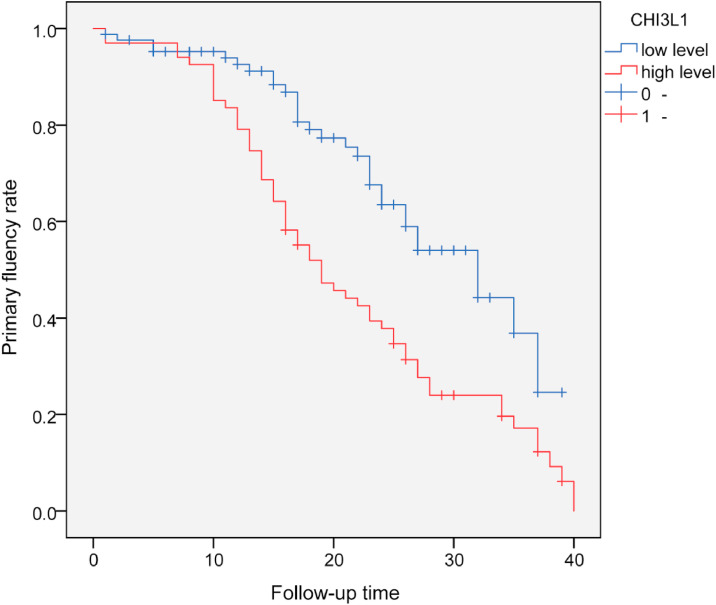
Kaplan-Meier survival analysis of serum CHI3L1 levels.

With serum CHI3L1 = 148.0 ng/mL as the cut-off value, all patients were divided into high-level group (≥ 148.0 ng/mL) and a low-level group (< 148.0 ng/mL). The primary fluency rate in the high-level group was significantly lower than that in the low-level group. Log-Rank test showed that there was a statistically significant difference in the primary fluency rate of AVF between the two groups without dysfunctional AVF (*p* < 0.001), suggesting that CKDG5 patients with high levels of CHI3L1 were more likely to develop dysfunctional AVF. The estimated time of postoperative dysfunctional AVF in the high CHI3L1 level group was 19-months (95 % CI: 14.6‒23.4), while the estimated time of postoperative dysfunctional AVF in the low CHI3L1 level group was 32-months (95 % CI 25.3‒38.8) (Fig. 3).

### Risk factors affecting dysfunctional AVF and construction of prediction model

**Fig. 4:**
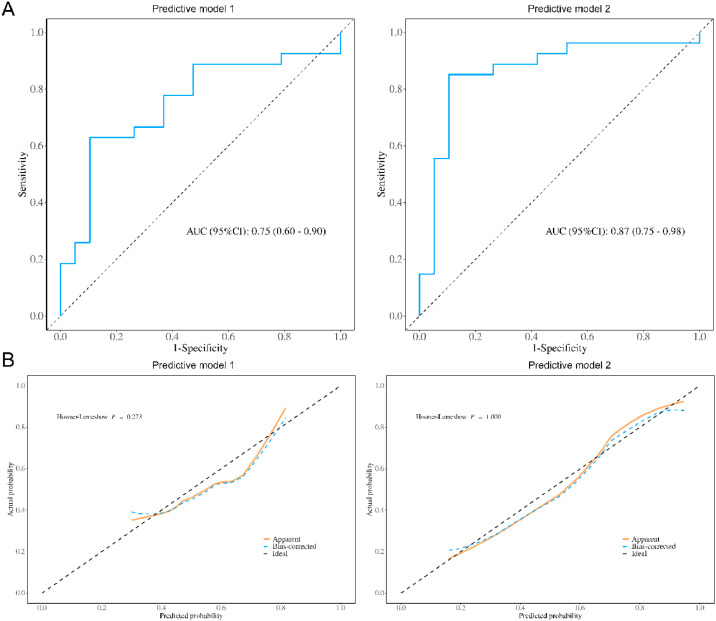
Multivariate logistic regression analysis model of dysfunctional AVF in CKDG5 patients. (A) ROC curve; (B) Model calibration curve (optimized). The HL test is the model fitting index, and the p value greater than 0.05 is considered to pass the HL test.

**Fig. 5:**
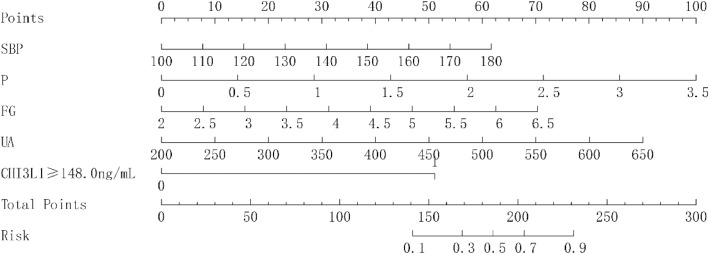
Nomogram of model prediction in CKDG5 patients.

Variables with significant differences (*p* < 0.05) in the univariate analysis of Table 1 were included in the multivariate logistic regression analysis (stepwise backward method). Multiple covariate diagnostics showed that the VIF for SBP, UA, FG, P, and CHI3L1 was 1.006, 1.070, 1.003, 1.083, and 1.029, respectively, with no covariance between the respective variables (VIF < 10). The clinical factors SBP, UA, FG, and P were used as a prediction Model 1. Prediction Model 2 included serum CHI3L1 on the basis of prediction Model 1. In addition, age and gender entered the model as calibration variables. As shown in Table 3, in the prediction Model 1, high levels of SBP, FG, P, and UA were independent risk factors for dysfunctional AVF postoperatively (*p* < 0.05). Prediction Model 2 confirmed that SBP, FG, P, UA, and elevated serum CHI3L1 were independent risk factors for dysfunctional AVF (*p* < 0.05). ROC curve analysis was performed on the two prediction models (Fig. 4A). The AUC of prediction Model 2 was higher than that of prediction Model 1 (*p* = 0.028). The accuracy of the model prediction was evaluated by drawing a calibration curve. Fig. 4B shows that the p-values of the two prediction models (HL test) were greater than 0.05, indicating that the models fitted well. The nomogram was employed to show the contribution of each variable in Model 2. In the nomogram, the corresponding scores of variables were obtained according to their values corresponding to the first line. The sum of the scores of the variables was the total score. The vertical axis of the total score corresponded to the probability of dysfunctional AVF (Fig. 5).

**Table 3:** Stepwise backward logistic regression analysis of risk factors for AVF dysfunction in CKDG5 patients.

Variables	Model 1		Model 2	
	OR (95 % CI)	p	OR (95 % CI)	p
Intercept	0.00 (0.00‒0.00)	<0.001	0.00 (0.00‒0.00)	<0.001
Systolic blood pressure	1.04 (1.01‒1.07)	0.01	1.04 (1.01‒1.07)	0.025
Phosphorus	3.46 (1.16‒10.36)	0.027	4.02 (1.14‒14.14)	0.03
Fibrinogen	2.04 (1.19‒3.51)	0.01	2.14 (1.13‒4.05)	0.199
Uric acid	1.01 (1.01‒1.01)	0.002	1.01 (1.01‒1.02)	0.003
Serum CHI3L1 ≥ 148.0 ng/mL			12.06 (3.92‒37.14)	<0.001

### DCA evaluation of model 2

**Fig. 6:**
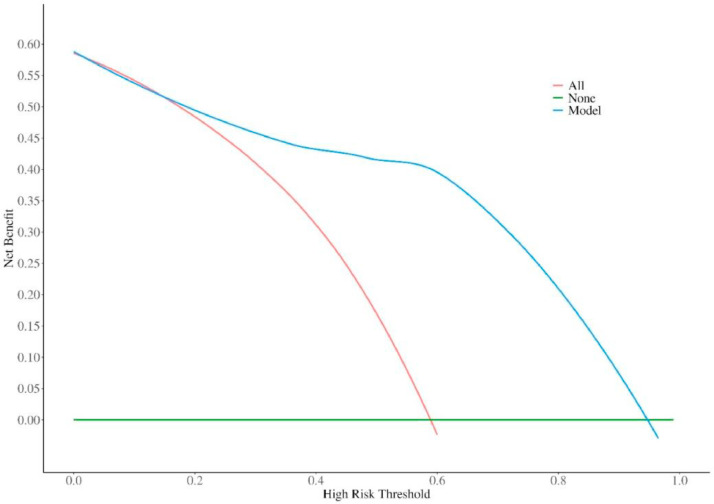
DCA curves of Model 2.

The red line in the figure is the decision curve of the model. When the threshold probability was 0.2‒0.9, the net benefit level of the nomogram was significantly higher, suggesting that the nomogram has good clinical applicability (Fig. 6).

## Discussion

Main findings: Serum CHI3L1 levels were significantly elevated in patients with CKDG5 and dysfunctional AVF. Multifactorial logistic regression and ROC curve analysis confirmed that CHI3L1 was an independent predictor of AVF dysfunction. A prediction model that combined CHI3L1 with SBP, WBC, FG, and P showed higher predictive accuracy compared with a baseline model that excluded CHI3L1. Column line graph visualization and decision curve analysis supported the clinical applicability of the model for identifying high-risk patients.

In patients undergoing MHD, vascular access is the key to effective dialysis. AVF has become the preferred vascular access for patients undergoing MHD, because of its low infection rate, few complications, and convenient use.^13^ In clinical practice, dysfunctional AVF is usually defined due to insufficient blood flow to meet the requirements of hemodialysis. As dialysis progresses, AVF also tends to fail. In the event of AVF failure, hemodialysis cannot be completed effectively, and long-term accumulation of waste in the body can cause serious complications. There are many factors leading to thrombosis and vascular stenosis in AVF.^8^^,^^14^ Early studies have proposed several predictors associated with postoperative fluency of AVF, such as traditional risk factors, such as age, gender, hypertension, diabetes, and hyperlipidemia, or non-traditional risk factors such as oxidative stress, inflammation, vascular calcification, uremic toxins, and endothelial cell dysfunction.^15^, ^16^, ^17^, ^18^ However, the risk of dysfunctional AVF varies greatly in each dialysis patient. Constructing and validating a model to accurately predict individual AVF fluency rates is crucial for identifying high-risk patients with dysfunctional AVF and facilitating early intervention.

Vascular inflammation is the key pathological process of atherosclerosis and vascular stenosis, and its core mechanism involves the abnormal expression and activity of various inflammatory mediators. It has been found that vascular smooth muscle cells induced by high cholesterol can secrete CHI3L1.^19^ CHI3L1 can induce atherosclerosis in human umbilical vein endothelial cells by regulating lipopolysaccharide.^20^ CHI3L1 knockout significantly inhibits atherosclerosis development.^13^ Direct knockout of CHI3L1 can inhibit the development of atherosclerotic plaques and vascular inflammation.^21^ CHI3L1 is therefore crucial to atherosclerosis and vascular stenosis.

This study first compared the differences between CKDG5 patients with and without dysfunctional AVP. CKDG5 patients with dysfunctional AVF had abnormal blood routine and blood biochemical indicators, such as P, Ca, PTH, etc. At the same time, it was also observed that CHI3L1 increased in CKDG5 patients with dysfunctional AVF, suggesting that it may have a certain correlation with dysfunctional AVF, and may predict and evaluate dysfunctional AVF. Subsequent multivariate logistic regression analysis and ROC curve analysis also confirmed the point of view of this study. In terms of molecular mechanism, CHI3L1 may cause thrombosis and stenosis of AVF through MCP-1, LPS and inflammation in MHD patients with repeated vascular endothelial injury and systemic microinflammation due to circulating metabolic waste, and ultimately lead to dysfunctional AVF.^11^ Liang et al. first proposed an independent association between serum CHI3L1 level and dysfunctional AVF.^10^ Similar to this study, our study found that in CKDG5 patients with high CHI3L1 levels, dysfunctional AVF occurred significantly earlier than in CKDG5 patients with low CHI3L1 levels. To better evaluate the prognosis of CKDG5 patients, this study screened out the most relevant factors for dysfunctional AVF: SBP, WBC, FG, P by stepwise backward logistic regression analysis. Serum P is associated with dysfunctional AVF.^22^ Elevated serum P or P overload may directly promote vascular injury, including vascular calcification, arterial stiffness, endothelial dysfunction, and left ventricular hypertrophy.^23^ Further, high serum P levels are associated with poor vascular access survival.^24^ Hyperphosphatemia is an independent risk factor for dysfunctional AVF (25 Our results revealed that CKDG5 patients with dysfunctional AVF had higher blood P levels. Elevated FG levels are an independent cardiovascular risk factor[26] and lead to increased coagulation activity, vascular endothelial dysfunction, resulting in vascular fistula dysfunction.^27^ Although previous studies have not confirmed the direct relationship between AVF and UA, several studies have shown that high UA is an independent risk factor for atherosclerosis.^28^, ^29^, ^30^ The hardened artery, reduced vascular elasticity, limited vasodilation, and stenosis can easily lead to dysfunctional AVF. In addition, SBP variability in MHD patients is an independent risk factor for AVF dysfunction and cerebrovascular accident. Therefore, the authors used four factors: SBP, WBC, FG, and P to construct a prediction Model 1. Based on this model, serum CHI3L1 was added to construct Model 2. The predictive value of the two models was compared, and the predictive value of Model 2, incorporating serum CHI3L1, was better than that of Model 1. The authors visualized the model through a nomogram, and the DCA showed that the nomogram of the model had good clinical applicability.

However, there are still limitations in this study. First of all, this study lacks some factors related to the fluency rate of AVF, such as vasodilation ability, vascular calcification, and inflammatory factors. The lack of this information may bias our results. Secondly, this study is a single-center small-sample-size study. The construction and verification of our model are limited to CKDG5 patients in our center, which means that the results of this study may be affected by selection bias, and the stability of the model is only tested by internal verification, without external verification.

## Conclusion

In this study, by comparing the serological indexes of CKDG5 patients with and without dysfunctional AVF, it was concluded that serum CHI3L1 level has a good value in predicting dysfunctional AVF in CKDG5 patients receiving MHD. Serum CHI3L1, SBP, P, WBC, and UA are independent influencing factors of dysfunctional AVF in CKDG5 patients. The risk prediction model of dysfunctional AVF in CKDG5 patients based on the above influencing factors has good clinical applicability. The visual nomogram of the model can be used as an effective tool for clinical prediction of the risk of dysfunctional AVF in CKDG5 patients. The prediction model in this study can assist the clinic in rapidly identifying MHD patients at high risk of dysfunctional AVF and guiding personalized interventions to prolong AVF lifespan. In the future, multi-center large sample validation is needed to optimize the model stability and explore CHI3L1-targeted intervention strategies to promote the transformation of the model into a clinical decision-making tool.

## Ethics approval

The present study was approved by the Ethics Committee of Qinghai Provincial People’s Hospital (n° 201806QH2544) and written informed consent was provided by all patients prior to the study start. All procedures were performed in accordance with the ethical standards of the Institutional Review Board and The Declaration of Helsinki, and its later amendments or comparable ethical standards.

## Funding

Not applicable.

## Data availability statement

The data must be requested from the corresponding author.

## CRediT authorship contribution statement

**You Wen Lin:** Conceptualization, Investigation, Writing – original draft. **Qing Zhang:** Conceptualization, Investigation, Writing – original draft. **Ying Sheng Xu:** Formal analysis, Validation. **Ting Qu:** Formal analysis, Writing – review & editing.

## Declaration of competing interest

The authors declare no conflicts of interest.
